# Global Screening of LUBAC and OTULIN Interacting Proteins by Human Proteome Microarray

**DOI:** 10.3389/fcell.2021.686395

**Published:** 2021-06-28

**Authors:** Lijie Zhou, Yingwei Ge, Yesheng Fu, Bo Wu, Yong Zhang, Lei Li, Chun-Ping Cui, Siying Wang, Lingqiang Zhang

**Affiliations:** ^1^Department of Physiopathology, Anhui Medical University, Hefei, China; ^2^State Key Laboratory of Proteomics, National Center for Protein Sciences (Beijing), Beijing Institute of Lifeomics, Beijing, China

**Keywords:** human proteome microarray, LUBAC, OTULIN, linear ubiquitination, LAG3

## Abstract

Linear ubiquitination is a reversible posttranslational modification, which plays key roles in multiple biological processes. Linear ubiquitin chain assembly complex (LUBAC) catalyzes linear ubiquitination, while the deubiquitinase OTULIN (OTU deubiquitinase with linear linkage specificity, FAM105B) exclusively cleaves the linear ubiquitin chains. However, our understanding of linear ubiquitination is restricted to a few substrates and pathways. Here we used a human proteome microarray to detect the interacting proteins of LUBAC and OTULIN by systematically screening up to 20,000 proteins. We identified many potential interacting proteins of LUBAC and OTULIN, which may function as regulators or substrates of linear ubiquitination. Interestingly, our results also hint that linear ubiquitination may have broad functions in diverse pathways. In addition, we recognized lymphocyte activation gene-3 (LAG3, CD223), a transmembrane receptor that negatively regulates lymphocyte functions as a novel substrate of linear ubiquitination in the adaptive immunity pathway. In conclusion, our results provide searchable, accessible data for the interacting proteins of LUBAC and OTULIN, which broaden our understanding of linear ubiquitination.

## Introduction

Ubiquitination is a reversible posttranslational modification and plays crucial roles in the regulation of various cellular pathways, such as the cell cycle, DNA damage repair, immune signaling, and diverse signal transduction (Komander and Rape, 2012; Swatek and Komander, 2016). Ubiquitination is an enzymatic cascade catalyzed by ubiquitin-activating enzyme E1, ubiquitin-conjugating enzyme E2, and ubiquitin ligase E3. Moreover, the substrates can be modified by mono-ubiquitination or poly-ubiquitination at lysine or non-lysine residues, such as serine, threonine, and cysteine (Cadwell, 2005; Shimizu et al., 2010; Swatek and Komander, 2016; Wang et al., 2017; Pao et al., 2018). Poly-ubiquitination occurs by diverse ubiquitin chain linkage *via* the formation of isopeptide bonds at the seven lysine sites of proximal ubiquitin. In addition, the first methionine (M1) of ubiquitin can also be modified by linking to another ubiquitin molecule *via* a peptide bond named linear ubiquitination or M1 ubiquitination (Kirisako et al., 2006; Spit et al., 2019).

Linear ubiquitination is a distinct linkage type of poly-ubiquitination, as the formation and erasure are catalyzed by unique enzymes named linear ubiquitin chain assembly complex (LUBAC) and OTULIN, respectively (Kirisako et al., 2006; Keusekotten et al., 2013). LUBAC is an enzyme complex of 600 kDa and contains three members: HOIP (RNF31), HOIL-1L (RBCK1), and SHARPIN (SIPL1) (Kirisako et al., 2006; Gerlach et al., 2011; Ikeda et al., 2011; Tokunaga et al., 2011). HOIP and HOIL-1 are both RING-in-between-RING (RBR) E3 ligases (Eisenhaber et al., 2007), but only HOIP catalyzes peptide bond formation between ubiquitin molecules *via* the RBR-LDD (linear ubiquitin chain determining domain) domain (Smit et al., 2012). HOIP alone has negligible catalysis activity. The UBL (ubiquitin-like) domain of HOIL-1L and SHARPIN directly binds to the UBA (ubiquitin-associated domain) of HOIP, which greatly boosts the activity of HOIP and promotes the formation of linear ubiquitin chains (Yagi et al., 2012; Fujita et al., 2018). OTULIN is a member of ovarian tumor (OTU) deubiquitinases. It is broadly accepted that OTULIN has exclusive cleavage activity towards linear ubiquitin chains, and OTULIN restricts LUBAC functions in an enzyme activity-dependent manner (Heger et al., 2018).

Linear ubiquitination, formed by LUBAC, is involved in canonical nuclear factor-κB (NF-κB) activation and the TNFR1 signaling complex (TNF-RSC) (Haas et al., 2009; Iwai and Tokunaga, 2009; Tokunaga et al., 2009; Niu et al., 2011). Deficiencies in *Hoip* (Peltzer et al., 2014), *Hoil-1l* (Peltzer et al., 2018), and *Sharpin* in mice have remarkable phenotypes in inflammation and immunity (HogenEsch et al., 1993; Seymour et al., 2007; Gerlach et al., 2011; Ikeda et al., 2011; Tokunaga et al., 2011). However, to make things complicated, *Otulin^*C*129*A/C*129*A*^* knock-in mice are embryonically lethal, and the TNF signal pathway is disordered (Heger et al., 2018). Further evidence indicates that OTULIN is also indispensable for LUBAC to function correctly (Elliott et al., 2014; Schaeffer et al., 2014). In addition, HOIL-1L catalyzes mono-ubiquitination at multiple LUBAC sites and attenuates LUBAC functions (Fuseya et al., 2020). Consequently, LUBAC regulation and linear ubiquitination are complicated and merit further study.

To date, limited numbers of substrates and regulators of linear ubiquitination have been reported. Tandem ubiquitin-binding entities (TUBEs) are useful tools to pull down ubiquitin chains, but they are limited in their affinity and specificity (Hjerpe et al., 2009). The accessible linear ubiquitin antibodies are not workable for immunoprecipitation and mass spectrometry. Internally tagged ubiquitin without lysine was constructed to pull down linear ubiquitin, which recognized several new substrates in TNF pathways (Kliza et al., 2017). However, this exogenous ubiquitin mutation may enrich unexpected substrates beyond physiological background levels. Owing to the low abundance of linear ubiquitin chains in cells, the present methods that rely on mass spectrometry cannot easily distinguish authentic substrates from background noise.

To further understand the novel functions of linear ubiquitination, we used a human proteome microarray (Sjöberg et al., 2016) to identify new interacting proteins of LUBAC and OTULIN. Using relatively strict criteria, we identified 330 potential interactors of LUBAC and 376 potential interactors of OTULIN, of which 260 were shared. We selected proteins for validation, and the results confirmed that the system was stable and reliable. Furthermore, we confirmed that lymphocyte activation gene-3 (LAG3, CD223) is a new substrate of linear ubiquitination, which may provide new ideas to understand the novel function of linear ubiquitination in T cell immunity.

## Materials and Methods

### Protein Expression and Purification

OTULIN cDNA was cloned into the pET28a vector with an N-terminal 6xHIS tag. After transforming into BL21 (DE3) strain and selecting on LB agar plates supplemented with 50 μg/ml kanamycin, a single clone was picked and cultured in LB medium supplemented with 50 μg/ml kanamycin until the OD_600_ reached 0.6. The expression of OTULIN was induced with 0.4 mM isopropyl β-D-1-thiogalactopyranoside (IPTG) at 20°C for 10 h before harvesting.

The HOIP expression vector was constructed using the pCDH-CMV vector with an N-terminal 6xHis tag, and transfection was performed using polyethylenimine for transient expression in HEK 293T cells. Cells were harvested 48 h after transfection.

To purify the His-tagged proteins, cells were resuspended and lysed in buffer containing 20 mM sodium phosphate, pH 8.0, 300 mM NaCl, 20 mM imidazole, and 0.5% Triton X-100. Lysozyme (20 μg/ml) and PMSF (0.5 mM) were added to the bacterial cell lysates. A protease inhibitor cocktail (Topscience, China) was added to the HEK 293T cell lysates. After sonication, the cell lysates were centrifuged at 15,000*g* for 15 min, and the insoluble pellet was discarded. The supernatant was incubated with His-tag Purification Resin (Beyotime, China) for the duration indicated by the manufacturer, and the resin was washed with lysis buffer five times to remove the uncoupled proteins. The His-tagged proteins were eluted with lysis buffer containing 200 mM imidazole, pH 7.5, and were dialyzed against phosphate-buffered saline (PBS; 20 mM sodium phosphate, pH 7.5, 150 mM NaCl). Protein purity was validated by Coomassie brilliant blue staining.

### Cell Culture

HEK 293T cell lines were cultured in Dulbecco’s modified Eagle’s medium high glucose (Hyclone, United States) supplemented with 10% fetal bovine serum (Gemini Bio, United States). All the culture media were supplemented with 100 U/ml penicillin and 0.1 mg/ml streptomycin. Cells were cultured at 37°C with 5% CO_2_.

### Plasmids and DNA Transfection

cDNAs for human HOIP, HOIL-1L, and OTULIN were amplified by reverse transcription from HEK 293T cells and inserted into the pFlag-CMV2 vector. Non-tagged ubiquitin, Myc-HOIP, and Myc-HOIP-CS (C699S/C702S/C871S/C874S) were constructed using Gibson assembly methods. LAG3 cDNA was gifted from Dr. Xiaoming Yang (State Key Laboratory of Proteomics, Beijing), and the mammalian expression vectors and the LAG3 mutations were constructed by PCR and Gibson assembly into pCMV-Myc and pCDNA3.1-Myc-His A.

cDNAs for ABI1, ABI2, SIRT3, SIRT5, DDX6, and WWP2 were amplified from human spleen cDNA and inserted into the pCMV-Myc vector.

Transfection was performed using polyethylenimine according to the standard protocol and cultured before harvesting for experiments.

### Co-immunoprecipitation

Cells were lysed in buffer containing 50 mM Tris-HCl, pH 7.4, 150 mM NaCl, 5 mM EDTA, and 1% Triton X-100 with protease inhibitor cocktail on ice before sonicating for 1 min. The lysates were centrifuged at 12,000*g* for 10 min, and the supernatants were transferred to 1.5-ml EP tubes and precleared with protein A/G agarose (Santa Cruz Biotechnology, United States) for 30 min at 4°C. Next, the lysates were incubated with specific antibodies for at least 1 h and then sequentially incubated with protein A/G agarose on a rotor at 4°C overnight. The agarose beads were washed four times with lysis buffer before boiling in Laemmli sample buffer, and the proteins were analyzed by immunoblotting.

### Immunoprecipitation, Linear Ubiquitination Assay, and Immunoblotting

Cells were lysed in buffer containing 50 mM Tris-HCl, pH 7.4, 150 mM NaCl, 5 mM EDTA, 0.5% sodium deoxycholate, and 1% Triton X-100 with protease inhibitor cocktail on ice. To detect linear ubiquitination, sodium dodecyl sulfate (SDS, 0.5%) was added to the cell lysates, which were then heated at 90°C for 5 min. The lysates were sonicated for 1 min, diluted to 0.1% SDS, and precleared for 30 min before incubating with the antibody and protein A/G agarose on a rotor at 4°C overnight. After washing four times with lysis buffer, Laemmli sample buffer was added, and the samples were boiled for 8 min. The samples were separated by SDS-PAGE and transferred onto a nitrocellulose membrane. The membranes were blocked in 5% non-fat milk for 1 h at room temperature and incubated with the following antibodies: anti-DDDDK tag (MBL, Japan), anti-Myc tag (MBL, Japan), anti-HA tag (MBL, Japan), anti-HIS tag (Biodragon, China), and anti-linear ubiquitin (Lifesensors, clone LUB9, United States). After incubation, the membrane was washed with 0.1% Tween-20 (TBST) buffer and incubated with a secondary antibody (Jackson, United States) or light-chain-specific secondary antibody (Abbkine, China) for 1 h at room temperature. After an additional wash with TBST, the membranes were incubated with enhanced chemiluminescence substrates (Thermo Fisher Scientific, United States) and developed in the darkroom.

### Immunofluorescence

HEK 293T cells were cultured in a 35-mm dish with a glass bottom and transfected with Flag-HOIP, Flag-OTULIN, and Myc-LAG3. The cells were washed with cold PBS 48 h after transfection and fixed with 4% paraformaldehyde for 15 min, permeabilized by 0.5% Triton X-100 for 20 min, and then blocked with 2% BSA for 30 min at room temperature. The cells were incubated with anti-DYKDDDDK antibody (Cell Signal Technology, United States) and anti-Myc antibody in 0.5% BSA at 4°C overnight. After incubation, the cells were washed four times with PBS containing 0.05% Tween-20 (PBST) for 20 min and then incubated with DAPI (Cell Signal Technology, United States) and Alexa Fluor 594 goat anti-rabbit IgG (Invitrogen, United States) or Alexa Fluor 488 goat anti-mouse IgG (Invitrogen, United States). Confocal images were visualized on a Nikon A1R confocal microscope.

### Human Proteome Microarray

**FIGURE 1:**
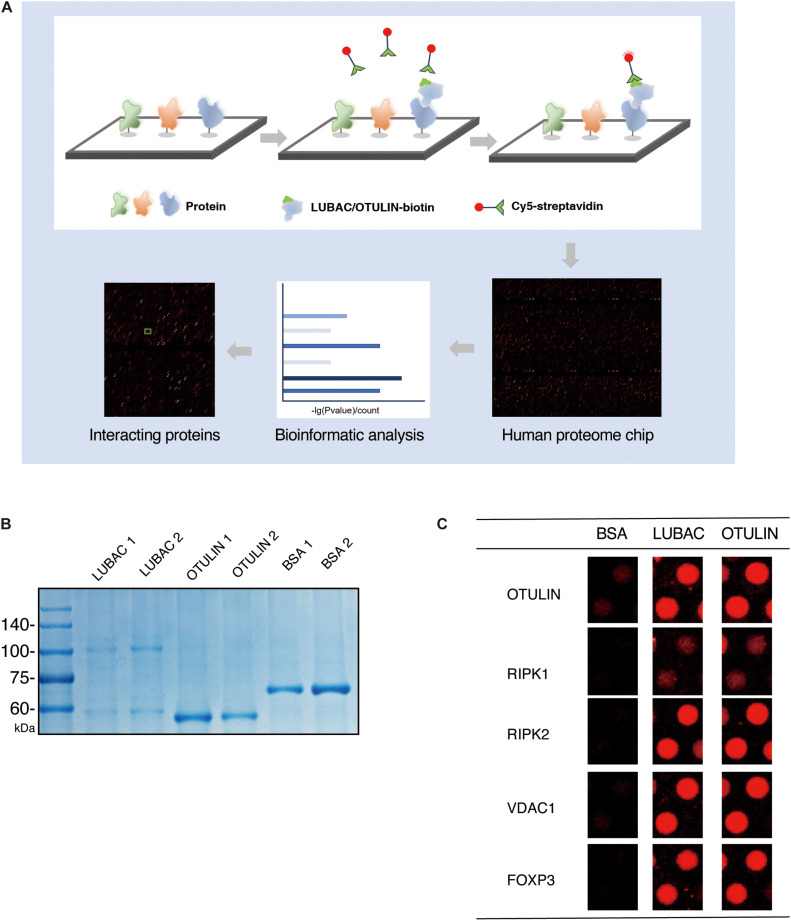
HuProt human proteome chip screening of LUBAC and OTULIN potentially interacting proteins. **(A)** Process diagram of the proteome microarray strategy. **(B)** Coomassie brilliant blue staining of purified LUBAC and OTULIN. **(C)** Five positive interacting proteins compared with the BSA control are shown.

We performed the human proteome microarray assays according to the HuProt User Guide (Figure 1A). The recombinant OTULIN and LUBAC proteins were labeled with biotin (Full Moon Biosystems, United States). Briefly, the microarrays were blocked with blocking buffer (PBS, 5% BSA, 0.1% Tween-20) and incubated with 5 μg/5 ml biotin-labeled protein sample for 1 h at room temperature with gentle shaking. The microarrays were washed four times with PBST and then incubated with 0.1% Cy5-streptavidin solution for 20 min at room temperature. After four PBST washes and three ddH_2_O washes, the desiccated microarrays were scanned with a GenePix 4000B (Axon Instruments, United States) at 635 nm. The data were extracted using GenePix Pro version 6.0 (Axon Instruments, United States).

### Protein Microarray Data Analysis

Data normalization was performed according to the HuProt User Guide.

*I* is the intensity of spot-normalized fluorescence signal at 635 nm, and *M* is the median of *I* of all spots across each microarray. The *Z*-score was calculated according to standard deviation (SD) as the standardized value of each spot [*Z*-score = (*I* - *M*)/SD].

When *I*_*mean*_ was the mean value of each protein spot, *I*_*Mean_Ratio*_ was the ratio of each spot and was used to filter the false and the positive spots in the negative control microarray (BSA).

The criteria used to filter the positive spots were *Z*-score ≥ 3 and *I*_*Mean_Ratio*_ ≥ 1.4, which were stringent, resulting in only almost 1.5% of proteins being isolated from the microarray.

## Results

### Screening of LUBAC and OTULIN Interacting Proteins by Human Proteome Microarray

To identify LUBAC and OTULIN interacting proteins *via* the ProtoArray Human Proteome Microarray, we first purified HOIP and OTULIN proteins *in vitro*. OTULIN expresses as a soluble protein in *Escherichia coli*, but the purification of HOIP is troublesome in prokaryotic expression systems. Using HEK 293T cells as the protein expression system, HIS-tagged HOIP and the LUBAC subunit HOIL-1 were successfully purified from the soluble cell lysate. Protein purity was confirmed by Coomassie brilliant blue staining (Figure 1B).

Using BSA as a negative control, the purified LUBAC and OTULIN proteins were labeled with biotin and then incubated with the proteome microarray (Supplementary Figure S1). Cy5-streptavidin was used to conjugate the biotin-labeled proteins, which directly interacted with the proteins in the microarray. After screening with the GenePix 4200B fluorescence microarray scanner, GenePix Pro version 6.0 was used to analyze the fluorescence signal. To validate the reliability of this assay, the spots of several proteins reported to interact with LUBAC or OTULIN (RIPK1, RIPK2, FOXP3, VDAC1, and OTULIN) were picked (Haas et al., 2009; Fiil et al., 2013; Elliott et al., 2014; Schaeffer et al., 2014; Kliza et al., 2017; Zhu et al., 2018). As shown in Figure 1C, these spots showed strong signals compared to the BSA control. These results validated the effectiveness of the human proteome microarray screening to detect the interactors of LUBAC and OTULIN.

### Verification of LUBAC and OTULIN Potential Interacting Proteins

**FIGURE 2:**
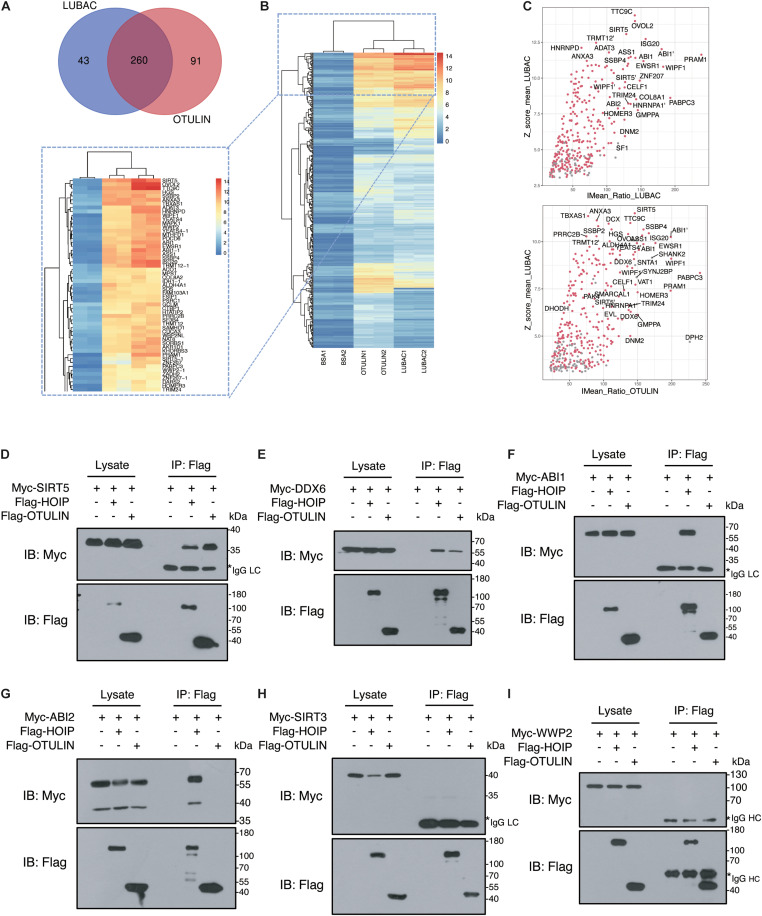
Verification of LUBAC and OTULIN potential interacting proteins. **(A)** Interacting proteins shared by LUBAC and OTULIN. **(B)** The signal strength of candidates is displayed in the heat map. **(C)** Scatter plots of potential interacting proteins for LUBAC and OTULIN. The shared candidates were marked as red dots, and the candidates detected by LUBAC or OTULIN alone were in gray. **(D–I)** Co-immunoprecipitation analysis of interactions between Myc-tagged candidates and Flag-HOIP and Flag-OTULIN in HEK 293T cells.

To narrow the number of spots filtered from the microarrays, we used the criteria *Z*-score ≥ 3 and *I*_*Mean_Ratio*_ ≥ 1.4. These criteria were relatively stringent, and only 330 proteins for LUBAC and 376 proteins for OTULIN were identified from the 20,000 proteins in the microarray. Interestingly, 260 of these proteins were co-interactors of LUBAC and OTULIN (Table 1, Figure 2A and Supplementary Tables S1-S3). A heat map was drawn to rank and visualize the co-interactors using the pheatmap package in R (Figure 2B). To visualize the potential interactors, these proteins are shown in the scatter plot and distributed with *I*_*Mean_Ratio*_ as well as *Z*-score_mean (Figure 2C). We constructed expression vectors for several of the top-ranked proteins with Myc tags, and a co-immunoprecipitation (Co-IP) assay confirmed that SIRT5 and DDX6 interact with LUBAC and OTULIN (Figures 2D,E). ABI1 and ABI2 interact with HOIP, but not OTULIN (Figures 2F,G). SIRT3 and WWP2 did not interact with either HOIP or OTULIN (Figures 2H,I). These results also suggest that, although the protein microarray data appear reliable, they are a mixed bag and merit further validation.

**TABLE 1:** The list of interacting proteins shared by LUBAC and OTULIN.

Protein	Protein (continued)	Protein (continued)	Protein (continued)	Protein (continued)
A1CF	CSTF2T	IGHG1	PAK4	SMARCE1
ABCA8	CTBP1	IGKC	PCBP4	SMPD1
ABI1	CTBP2	IRF2BP1	PDCD6	SOHLH2
ABI2	CUTA	IRF2BP2	PFKP	SORBS1
ACO1	CYB5R1	Irx5	PNKP	SORBS3
ACOT7	DARS2	ISCU	POGZ	SORD
ACSL6	DCX	ISG20	POP7	SOX6
ADAMTSL4	DDX6	ITPKB	PPP1R13L	SPATC1
ADAT3	DECR2	IVD	PRAM1	SPRR4
AKAP8	DHODH	KCNAB1	PRR30	SRA1
AKR1C3	DLG3	KCNAB2	PRR35	SRRT
AKR1D1	DNALI1	KDM1A	PRRC2B	SRXN1
ALDH16A1	DNM2	KHDRBS1	PSMB4	SSBP1
ALDH4A1	DOK1	KHDRBS3	PSRC1	SSBP2
ALKBH2	DTX2	KIF23	PTK2	SSBP4
ALKBH3	ECI2	KLHDC9	PUF60	STARD7
AMBRA1	EIF4G3	LAG3	PXK	STAU2
AMOTL2	EIF4H	LARS2	PYCR2	SULT1B1
ANGPTL2	ELAVL1	LNP	PYCRL	SULT1C2
ANXA3	ELAVL2	LOC105372481	QARS	TAF6
APTX	ELAVL4	LONP1	QKI	TAF9B
ARPC1B	ELN	LOR	RAB2B	TBXAS1
ARPC3	ENAH	MAGEB1	RAB5A	TCF7L1
ASS1	EVL	MAPK1	RAB5C	TIA1
ATIC	EWSR1	MAPK3	RALY	TK1
BAG6	F2	MBNL3	RBM12	TLE3
BC014212	FAAH2	MBP	RBM3	TMEM116
BC035666	FAM103A1	MCCC2	RBM42	TRIM24
BC047522.1	FAM120B	MCM7	RBM46	TRMT12
BCAR3	FAM49B	MIF	RBMS1	TRMT2A
BCS1L	FAM81A	MISP	RBMS2	TST
BLVRB	FKBP1A	MPST	RPL30	TTC9
BPHL	FOXP4	MSI2	RPLP0	TTC9C
C11orf1	FSCB	MTHFD1	RPP25	TTLL1
C17orf82	FSIP1	NABP1	RTCA	TUFM
C1orf74	FUBP1	NAT6	RXRA	UNG
C1orf94	GAPDH	NCOA3	SAMD4B	VASP
C21orf59	GBGT1	NECAP2	SAMHD1	VAT1
C9orf9	GCLM	NFYC	SATB1	WBP2NL
CBLN4	GMPPA	NG_006966.3	SCEL	WIPF1
CCNB1IP1	GPT2	NME2	SDS	WWP2
CDCA3	GSTZ1	NTPCR	SF3B4	XAGE3
CELF1	GTF2B	NUDT16L1	SGK494	XDH
COASY	HCFC2	NUDT6	SH3GLB2	XPNPEP3
COL8A1	HGS	NUMBL	SHMT1	XRN2
COL8A2	HNRNPA1	NUPL2	SIRT3	YAP1
CPT1A	HNRNPC	ODAM	SIRT5	YEATS4
CRY2	HNRNPD	OLA1	SKIL	ZADH2
CRYZ	HOMER3	OPHN1	SLC25A16	ZFYVE1
CSNK1G1	HSPD1	OVOL2	SLC30A6	ZNF207
CSRP1	HTATIP2	PABPC3	SLFN5	ZNF385A
CSRP3	IDH1	PABPC4	SMARCAL1	ZNF385B

### Bioinformatics Analysis of the Potential LUBAC and OTULIN Interacting Proteins

To gain further insight into the novel functions of LUBAC and OTULIN, we performed Gene Ontology (GO) enrichment analysis and pathway analysis with the shared interactors (Gene Ontology Consortium, 2004). We performed these analysis using the Database for Annotation, Visualization, and Integrated Discovery (Dennis et al., 2003).

**FIGURE 3:**
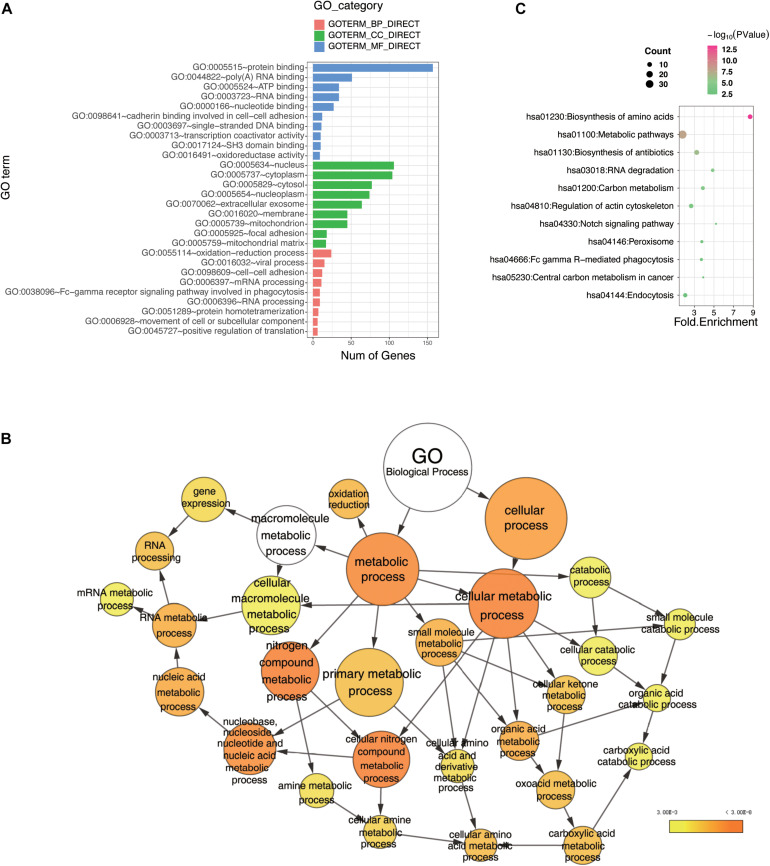
Bioinformatics analysis of the LUBAC and OTULIN potential interacting proteins. **(A)** Gene Ontology (GO) analysis showed the enrichment of potential interacting proteins of LUBAC and OTULIN in terms of GO categories BP, MF, and CC. Clustering based on information provided by Database for Annotation, Visualization, and Integrated Discovery database and visualized by bar plot in R. **(B)** GO analysis showed the enrichment of potential interacting proteins in terms of GO categories BP. The node size represents the gene number in the category, while the color change from yellow to orange indicates the change in *P*-value from large to small values for the corresponding category. Categorizations are based on information using the BiNGO plugin in Cytoscape. **(C)** Enriched pathways of potential interacting proteins analyzed by Kyoto Encyclopedia of Genes and Genomes. The node size represents the gene number in the corresponding pathway, while the color change from red to green indicates the change in *P*-value from large to small values for the corresponding pathway. These results were visualized by bubble chart in R.

Currently, our understanding of linear ubiquitination is mainly limited to inflammatory and immune signaling pathways. However, these pathways were not enriched in the top positions in our data. As shown in Figure 3A, the bar plot ranked the GO enrichment results in biology process (BP), cellular component (CC), and molecular function (MF). The results of the BP analysis showed that the potential interactors of LUBAC and OTULIN were enriched mainly in various metabolic processes and RNA processing. These results indicated that linear ubiquitination may have additional functions in the regulation of pre-translation level of proteins. For MF, the candidates were mostly classified into two groups: binding, including nucleic acid binding and nucleotide binding, and oxidoreductase activity. For CC, the candidates were enriched in the cytoplasm, membrane, and nucleus. These data showed that linear ubiquitination is involved in broad cellular biological processes, molecular functions, and interactions with proteins in different subcellular locations. To better visualize the GO enrichment results, we used the BiNGO plugin in Cytoscape to rebuild the enrichment results (Shannon et al., 2003; Maere et al., 2005). As shown in Figure 3B, the biology process is mainly clustered in metabolism, especially amino acid metabolism and catabolic process. The visualization of CC and MF is shown in Supplementary Figures S2B,C.

To further understand the signaling pathways of the LUBAC- and OTULIN-interacting proteins, we performed Kyoto Encyclopedia of Genes and Genomes pathway analysis (Kanehisa and Goto, 2000; Kanehisa, 2002), and the results were visualized by bubble chart in R. The interactors were predominantly enriched in 11 pathways, of which the top five were biosynthesis of amino acids, metabolic pathways, biosynthesis of antibiotics, RNA degradation, and carbon metabolism (Figure 3C).

Previously, our understanding of linear ubiquitination is subjected to immunity and inflammation, yet the ongoing research have uncovered the new functions in mitosis (Wu et al., 2019), viral infection (Zuo et al., 2020), protein quality control (Well et al., 2019), and regulation in diverse pathways. These data indicated that LUBAC, OTULIN, or linear ubiquitination may have broad functions beyond the present indications, which merit further exploration.

### LAG3 Harbors Linear Ubiquitination Mediated by HOIP

Previous evidence indicates that linear ubiquitination regulates T cell-mediated immunity, but the mechanism has been poorly elucidated (Ikeda, 2015; Shimizu et al., 2015). LAG3, an inhibitory lymphocyte receptor, was shown to be a potential interactor of LUBAC and OTULIN by our data. LAG3 is a type I transmembrane protein expressed on activated T cells and natural killer (NK) cells, consisting of four extracellular Ig-like domains (D1–D4) and several conserved motifs in the cytoplasmic segment (Triebel et al., 1990; Workman et al., 2002; Macon-Lemaitre and Triebel, 2005; Andrews et al., 2017; Zhang et al., 2017; Wang et al., 2019). The conserved cytoplasmic segment is indispensable for the inhibitory function of LAG3, but the downstream regulators and effectors have not been clearly described.

**FIGURE 4:**
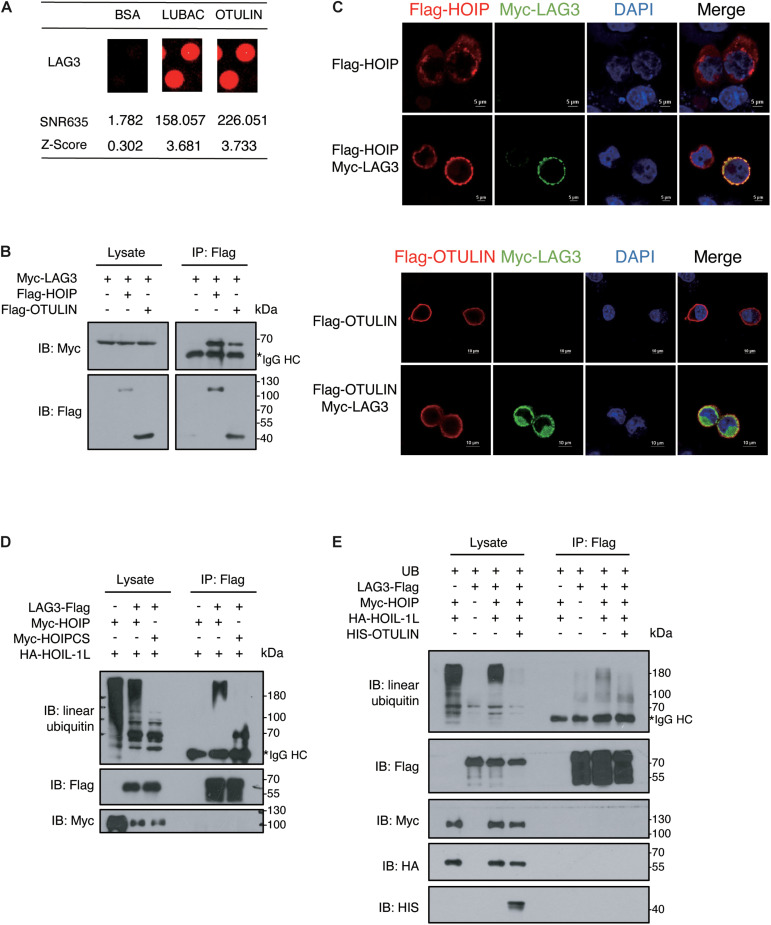
LAG3 harbors linear ubiquitination mediated by HOIP. **(A)** Potential interacting proteins of LAG3 are shown compared with the BSA control. **(B)** Co-immunoprecipitation analysis of interactions between Myc-LAG3 and Flag-HOIP and Flag-OTULIN in HEK 293T cells. **(C)** Immunofluorescence analysis of the interactions between Flag-HOIP, Flag-OTULIN, and Myc-LAG3 in HEK293T cells. **(D)** Immunoprecipitation analysis of linear ubiquitination of Flag-LAG3 in HEK293T cells co-transfected with HA-HOIL-1L and Myc-HOIP wild type or its catalytically inactivated mutants (C699S/C702S/C871S/C874S). **(E)** Immunoprecipitation analysis of overexpressing HIS-OTULIN decreases the linear ubiquitination of LAG3.

In our study, LAG3 was identified as a potential LUBAC and OTULIN interactor by comparing the signals with the negative BSA control (Figure 4A). To confirm the association of LAG3 with HOIP/OTULIN, exogenous co-immunoprecipitation and immunofluorescence assays were performed, which revealed that LAG3 interacts with HOIP/OTULIN in the cell (Figures 4B,C). Next, we sought to detect the linear ubiquitination of LAG3. Exogenous ubiquitination assays showed that HOIP coupled with HOIL-1L ubiquitinated LAG3, while the catalytically inactive HOIP mutant (HOIP-CS) did not ubiquitinate LAG3 (Figure 4D). In addition, overexpression of OTULIN greatly decreased the linear ubiquitination of LAG3 (Figure 4E).

**FIGURE 5:**
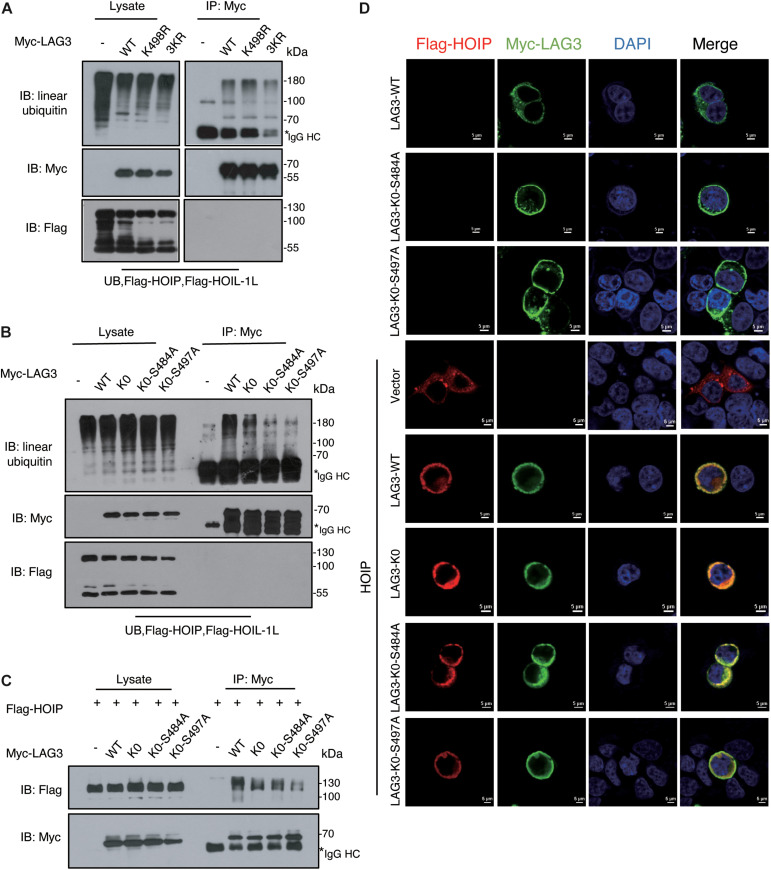
LAG3 can be linear-ubiquitinated at multiple sites by LUBAC. **(A)** Immunoprecipitation analysis of LAG3 ubiquitination in HEK293T cells co-transfected with Myc-LAG3 wild type or its mutants K498R and 3KR (3KR = K356R, K357R, and K398R). **(B)** Immunoprecipitation analysis of LAG3 ubiquitination in HEK293T cells co-transfected with Flag-LAG3 wild type, K0, K0-S484A, and K0-S497A (K0 = K21R/K297R/K356R/K366R/K498R). **(C)** Immunofluorescence analysis for the cellular location of LAG3 wild type and mutations, the interaction between Flag-HOIP and Myc-LAG3 wild type, and mutations in HEK293T cells. **(D)** Co-immunoprecipitation analysis of interactions between Myc-LAG3 wild type, mutations, and Flag-HOIP in HEK 293T.

LAG3 consists of five lysine residues, of which three are conserved (K356, K366, and K498). K498 is the only lysine residue in the cytoplasmic segment, and the KIEELE motif (498–503) is crucial for the inhibitory function of LAG3 (Workman et al., 2002). However, the K498R, 3KR (K356R/K366R/K498R), and LAG3-K0 mutations showed only a slight decline in linear ubiquitination (Figures 5A,B). Ubiquitination can be catalyzed at non-lysine residues, such as serine, threonine, or cysteine (Cadwell, 2005; McDowell and Philpott, 2013; Pao et al., 2018), and HOIL-1 can catalyze the formation of oxyester bonds between ubiquitin and serine or threonine in substrates (Kelsall et al., 2019; Fuseya et al., 2020). LAG3 contains two conserved serine residues (S484 and S497) in the intracytoplasmic tail, and previous results have indicated that phosphorylation is not involved in the inhibitory function of LAG3 (Bae et al., 2014). The ubiquitination assay showed that LAG3-K0-S484A and LAG3-K0-S497A mutations displayed obviously reduced linear ubiquitination compared with the K0 mutation (Figure 5B). Furthermore, exogenous co-immunoprecipitation and immunofluorescence assays confirmed that LAG3 mutations (K0, K0-S484A, and K0-S497A) still interact with HOIP in the cell, and the mutations had not altered the cellular localization of LAG-3 (Figures 5C,D). K498 of the KIEELE motif is indispensable for the negative functions of LAG3, and S497 is adjacent to the KIEELE motif. Our results show that multiple sites of LAG3 can be linear-ubiquitinated by LUBAC, and the redundant ubiquitination sites may be responsible for the regulatory functions of LAG3.

## Discussion

Linear ubiquitination is an important posttranslational modification that is involved in multiple biological processes. The ubiquitin ligase complex LUBAC, composed of HOIP, HOIL-1L, and SHARPIN, generates linear (M1)-linked polyubiquitin chains. The deubiquitinase OTULIN specifically disassembles linear ubiquitin chains. Currently, linear ubiquitination is known to regulate TNF-RSC and NF-κB signaling pathways to maintain inflammation and immune homeostasis, but our understanding of linear ubiquitination is limited. Our results showed that LUBAC and OTULIN have a broad landscape of interacting proteins, hinting that linear ubiquitination has additional functions beyond our present understanding.

The interactors of LUBAC and OTULIN were detected in the absence of a cellular model, circumventing the effects of the TNF and NF-κB signaling pathways in the cellular background. However, *in vitro* high-throughput microarray screening may be helpful to find new substrates and regulators of linear ubiquitination. Inevitably, the *in vitro* microarray assay neglects the subcellular location of the proteins, which has added to the false-positive ratio.

Using relatively stringent criteria, we identified 330 potential interacting proteins of LUBAC and 376 potential interacting proteins of OTULIN, of which 260 were shared. We used co-immunoprecipitation to verify the interaction of these potential interacting proteins with HOIP and OTULIN. These results indicated that our protein microarray data were reliable, and the positive rate was satisfactory.

Bioinformatics analysis revealed that the candidate proteins were enriched in several novel pathways, such as metabolic pathways, RNA processing, and biosynthesis processing. These results also indicate new functions of linear ubiquitination for exploration.

Furthermore, we verified that LAG3 is a new substrate of linear ubiquitination mediated by LUBAC. LAG3 is a transmembrane protein expressed on activated T cells and NK cells. LAG3 consists of conserved motifs in the cytoplasmic domain, which possesses two potential serine phosphorylation sites, “KIEELE” motif and “EP” repetitive motif. Interestingly, the LAG3 cytoplasmic motif does not have immunoreceptor tyrosine-based inhibition motifs or immunoreceptor tyrosine-based switch motifs, which are phosphorylation motifs found in many receptors that recruit tyrosine phosphatases to limit TCR signaling (Unkeless and Jin, 1997). Studies in recent years have suggested that LAG3 may have different regulatory mechanisms beyond phosphorylation. Our results suggest that the conserved KIEELE motif and the serine sites in the LAG3 intracellular segment can be ubiquitinated by LUBAC, indicating that ubiquitination, not phosphorylation, may be responsible for the inhibitory functions of LAG3. In addition, the role of linear ubiquitination in adaptive immunity has been poorly elucidated (Ikeda, 2015). The negative regulatory role of LAG3 in the T cell signaling pathway explains the phenotypes in the *Hoip^Δ*Cd*4^*, *Hoil^Δ*Cd*4^*, and *Cpdm* mice, which have a substantial reduction in the number of T cells and defective development and function of T cells (Park et al., 2016; Teh et al., 2016).

In summary, we have performed a global protein interaction screening of LUBAC and OTULIN using the human proteome microarray. Our results have broadened the LUBAC and OTULIN interactome and may serve as a valuable resource to explore new functions of linear ubiquitination.

## Data Availability Statement

All the data that support the conclusions are presented in this paper. The raw data for the human proteome microarray are provided in Supplementary Table 4.

## Author Contributions

LqZ and SW designed the research. LjZ and YG performed the research and wrote the manuscript. C-PC contributed to results analysis and discussion. YF, BW, LL, and YZ contributed new reagents and to the discussion. All authors contributed to the article and approved the submitted version.

## Conflict of Interest

The authors declare that the research was conducted in the absence of any commercial or financial relationships that could be construed as a potential conflict of interest.

## Supplementary Material

Supplementary Figure 1 The whole chip picture of BSA **(A)**, HIS-LUBAC **(B)**, and HIS-OTULIN **(C)**.

Supplementary Figure 2 Gene Ontology (GO) analysis showed the enrichment of potential interacting proteins in terms of GO categories molecular function **(A)** and cellular component **(B)**. The node size represents the gene number in the category, while the color change from yellow to orange indicates the change in *P*-value from large to small values for the corresponding category. The categorizations are based on information using the BiNGO plugin in Cytoscape.

Supplementary Table 1 Detailed list of potential interacting proteins shared by LUBAC and OTULIN.

Supplementary Table 2 Detailed list of potential interacting proteins of LUBAC.

Supplementary Table 3 Detailed list of potential interacting proteins of OTULIN.

Supplementary Table 4 Raw data for human proteome microarray.
