# Supplementary material for: Global Screening of LUBAC and OTULIN Interacting Proteins by Human Proteome Microarray
Source: Front Cell Dev Biol. 2021 Jun 28;9:686395. doi: 10.3389/fcell.2021.686395 (PMC8274477; doi:10.3389/fcell.2021.686395)
Supplement: Supplementary Figure 1 — The whole chip picture of BSA (A), HIS-LUBAC (B), and HIS-OTULIN (C). [file Image_1.pdf]

Supplementary Material

Supplementary Data

Supplementary Figure 1

Supplementary Figure 2

Supplementary Table 1: The detailed list of potential interacting proteins shared by LUBAC and OTULIN

Supplementary Table 2: The detailed list of potential interacting proteins detected by LUBAC alone

Supplementary Table 3: The detailed list of potential interacting proteins detected by OTULIN alone

Supplementary Table 4: The raw data for the human proteome microarray

Supplementary Figure 1

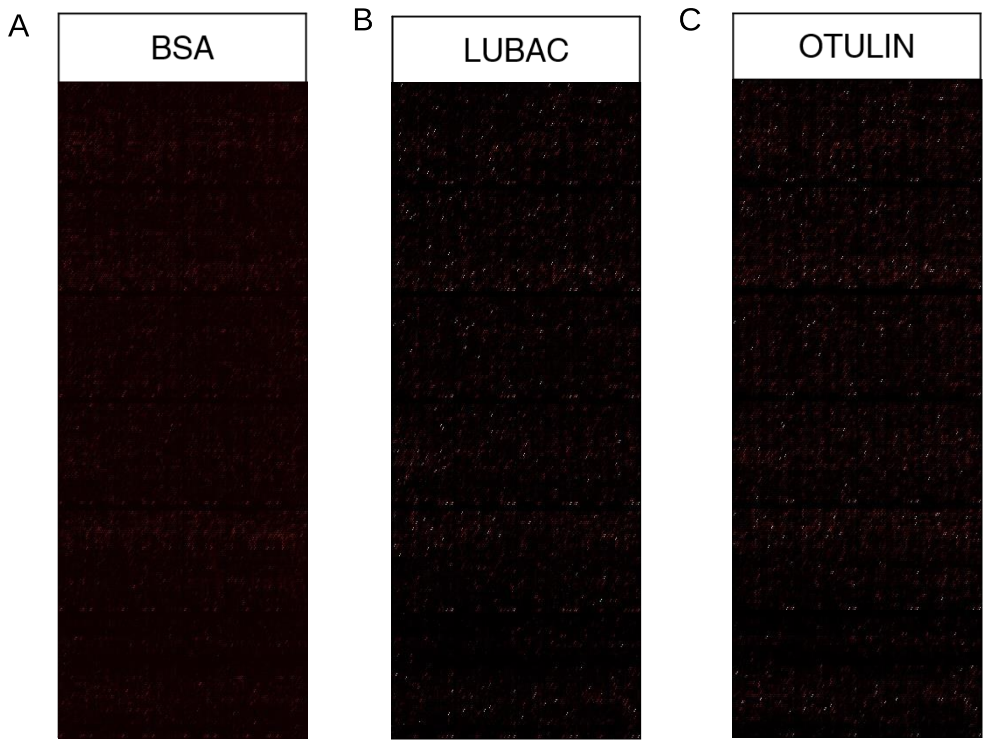

Supplementary Figure 1. The whole chip picture of negative group BSA(A), training group HIS-LUBAC(B) and training group HIS-OTULIN(C).
