# Supplementary material for: Global Screening of LUBAC and OTULIN Interacting Proteins by Human Proteome Microarray
Source: Front Cell Dev Biol. 2021 Jun 28;9:686395. doi: 10.3389/fcell.2021.686395 (PMC8274477; doi:10.3389/fcell.2021.686395)
Supplement: Supplementary Figure 2 — Gene Ontology (GO) analysis showed the enrichment of potential interacting proteins in terms of GO categories molecular function (A) and cellular component (B). The node size represents the gene number in the category, while the color change from yellow to orange indicates the change in P-value from large to small values for the corresponding category. The categorizations are based on information using the BiNGO plugin in Cytoscape. [file Image_2.pdf]

A

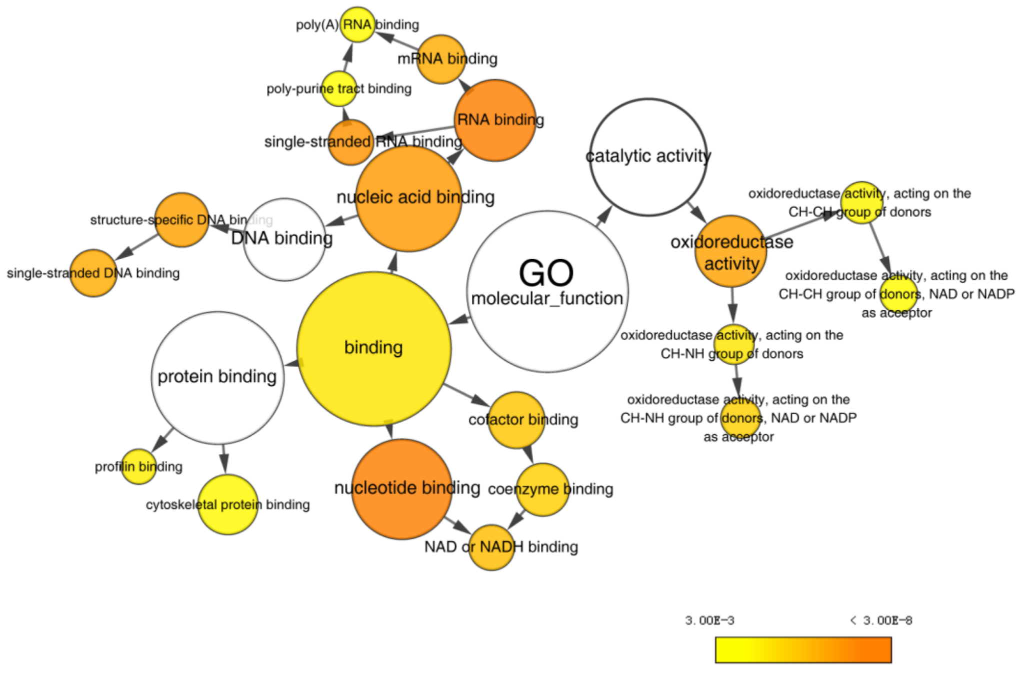

B

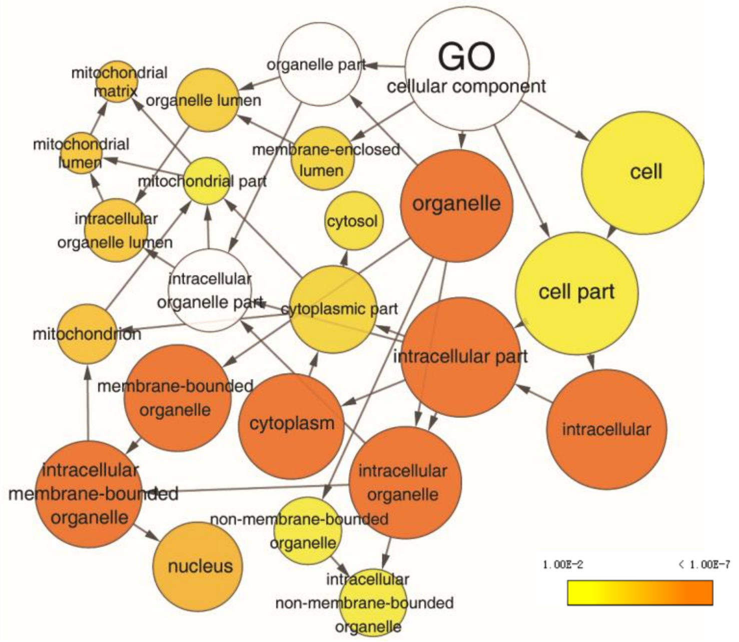

Supplementary figure 2

GO analysis showed enrichment of potential interacting proteins in terms of GO categories MF (A) and CC (B). The node size represents the gene number in the category, while the color change from yellow to orange indicates the change in the P-value from large to small values for the corresponding category. Categorizations are based on information using the BiNGO plugin in Cytoscape.
