# Supplementary material for: Global Screening of LUBAC and OTULIN Interacting Proteins by Human Proteome Microarray
Source: Front Cell Dev Biol. 2021 Jun 28;9:686395. doi: 10.3389/fcell.2021.686395 (PMC8274477; doi:10.3389/fcell.2021.686395)
Supplement: Supplementary Table 1 — Detailed list of potential interacting proteins shared by LUBAC and OTULIN. [file Table_1.docx]

Supplementary Material

# Supplementary Data

Supplementary Figure 1

Supplementary Figure 2

Supplementary Table 1: The detailed list of potential interacting proteins shared by LUBAC and OTULIN

Supplementary Table 2: The detailed list of potential interacting proteins detected by LUBAC alone

Supplementary Table 3: The detailed list of potential interacting proteins detected by OTULIN alone

Supplementary Table 4: The raw data for the human proteome microarray

## Supplementary Table 1

Supplementary Table 1: The detailed list of potential interacting proteins shared by LUBAC and OTULIN

| Name | Z-Score  (LUBAC) | Z-Score  (LUBAC) | IMean_Ratio  (LUBAC-BSA) | Z-Score  (OTULIN) | Z-Score  (OTULIN) | IMean_Ratio  (OTULIN-BSA) |
| --- | --- | --- | --- | --- | --- | --- |
| A1CF | 5.554 | 5.271 | 83.284 | 3.171 | 3.132 | 65.26726772 |
| ABCA8 | 6.462 | 7 | 89.623 | 4.499 | 4.563 | 79.64193816 |
| ABI1 | 11.448 | 11.374 | 141.81 | 9.795 | 9.939 | 158.410583 |
| ABI1' | 12.585 | 11.46 | 180.412 | 10.727 | 9.767 | 198.6174446 |
| ABI2 | 7.703 | 8.062 | 116.093 | 5.775 | 5.169 | 105.7169773 |
| ACO1 | 8.985 | 7.779 | 56.672 | 9.068 | 9.068 | 78.87654933 |
| ACOT7 | 4.736 | 4.273 | 23.564 | 4.21 | 3.978 | 28.02575125 |
| ACSL6 | 3.861 | 4.162 | 22.442 | 4.652 | 4.812 | 34.11918725 |
| ADAMTSL4 | 5.256 | 4.561 | 35.99 | 5.149 | 4.777 | 47.21423077 |
| ADAT3 | 11.753 | 11.831 | 102.988 | 8.772 | 8.772 | 99.40261438 |
| AKR1C3 | 4.552 | 4.296 | 48.863 | 4.167 | 4.388 | 61.57941176 |
| AKR1D1 | 11.222 | 10.568 | 77.487 | 9.256 | 9.32 | 85.41938226 |
| ALDH16A1 | 6.154 | 5.056 | 65.256 | 8.4 | 8.341 | 124.1487825 |
| ALDH4A1 | 8.778 | 8.837 | 74.616 | 10.56 | 9.478 | 108.9968172 |
| ALKBH2 | 5.76 | 6.092 | 57.337 | 6.005 | 6.012 | 75.22432437 |
| ALKBH3 | 4.259 | 3.507 | 28.645 | 3.714 | 3.561 | 35.14912994 |
| AMBRA1 | 3.642 | 3.404 | 53.744 | 3.415 | 3.784 | 71.60725352 |
| AMBRA1' | 6.437 | 5.819 | 72.725 | 6.678 | 6.291 | 99.3497191 |
| AMOTL2 | 5.306 | 5.606 | 102.415 | 4.68 | 5.317 | 122.219429 |
| ANGPTL2 | 4.78 | 5.028 | 52.784 | 3.9 | 4.45 | 58.94576588 |
| ANXA3 | 10.882 | 11.522 | 70.413 | 10.899 | 11.354 | 90.01441886 |
| APTX | 5.688 | 6.404 | 35.037 | 8.821 | 7.02 | 58.74812796 |
| ARPC1B | 6.563 | 6.167 | 47.155 | 5.633 | 5.581 | 54.06769106 |
| ARPC3 | 3.499 | 3.65 | 30.608 | 4.887 | 4.615 | 52.09638291 |
| ASS1 | 11.597 | 11.374 | 134.925 | 10.642 | 10.012 | 156.5417021 |
| ATIC | 3.727 | 4.072 | 20.749 | 7.379 | 7.414 | 49.55125 |
| BC014212 | 5.934 | 5.789 | 42.785 | 5.189 | 4.936 | 48.23172084 |
| BC035666 | 4.485 | 4.099 | 42.133 | 4.199 | 3.962 | 52.31012533 |
| BC047522.1 | 6.351 | 6.974 | 93.581 | 4.927 | 4.175 | 84.32886819 |
| BC047522.1' | 6.057 | 6.894 | 86.201 | 5.08 | 4.388 | 82.84913495 |
| BCAR3 | 4.292 | 4.087 | 44.513 | 3.444 | 3.582 | 49.21963603 |
| BCS1L | 3.878 | 3.987 | 53.401 | 3.434 | 3.859 | 64.89814494 |
| BLVRB | 6.693 | 6.429 | 67.116 | 9.451 | 9.586 | 124.1946975 |
| BPHL | 5.141 | 4.876 | 31.836 | 6.897 | 6.818 | 55.70834537 |
| C11orf1 | 4.03 | 4.186 | 43.074 | 4.432 | 3.516 | 54.39524934 |
| C17orf82 | 4.258 | 5.074 | 42.178 | 3.483 | 4.03 | 44.7827641 |
| C1orf74 | 5.775 | 6.082 | 30 | 5.462 | 5.426 | 35.82670428 |
| C1orf94 | 4.405 | 4.733 | 78.123 | 4.661 | 4.335 | 100.0845217 |
| C21orf59 | 4.089 | 3.198 | 30.383 | 5.334 | 4.578 | 52.8490399 |
| C9orf9 | 3.28 | 3.215 | 41.672 | 5.292 | 4.056 | 76.43073567 |
| CCNB1IP1 | 3.295 | 3.228 | 16.919 | 3.29 | 3.631 | 23.37011321 |
| CDCA3 | 9.79 | 10.308 | 63.484 | 8.232 | 8.335 | 67.81541076 |
| CELF1 | 9.112 | 9.586 | 126.017 | 7.142 | 7.724 | 130.1568252 |
| COASY | 6.332 | 5.771 | 34.551 | 7.691 | 7.711 | 56.33910114 |
| COL8A1 | 9.556 | 7.494 | 142.95 | 4.798 | 3.712 | 95.19626168 |
| COL8A2 | 9.957 | 7.995 | 50.922 | 8.898 | 8.39 | 63.29759742 |
| CPT1A | 4.781 | 4.114 | 29.051 | 5.001 | 4.812 | 41.4253212 |
| CRY2 | 5.124 | 5.935 | 63.332 | 6.298 | 5.997 | 90.78960684 |
| CRYZ | 7.041 | 6.863 | 86.41 | 9.256 | 9.866 | 151.8309622 |
| CSNK1G1 | 6.427 | 6.526 | 80.915 | 6.139 | 4.813 | 89.21115894 |
| CSRP1 | 3.091 | 3.125 | 40.924 | 5.065 | 4.425 | 79.31 |
| CSRP3 | 9.007 | 6.831 | 46.443 | 5.496 | 5.712 | 43.06748466 |
| CSTF2T | 7.618 | 6.474 | 45.532 | 3.499 | 3.27 | 29.5251113 |
| CTBP1 | 10.129 | 9.314 | 58.993 | 8.335 | 8.082 | 64.52644338 |
| CTBP2 | 4.372 | 4.185 | 33.543 | 4.191 | 4.221 | 42.96193548 |
| CUTA | 5.215 | 4.441 | 29.887 | 5.231 | 4.718 | 39.91970767 |
| CYB5R1 | 3.928 | 4.172 | 22.302 | 5.197 | 4.542 | 34.52081267 |
| DARS2 | 9.344 | 7.206 | 69.541 | 8.206 | 6.264 | 78.84368928 |
| DCX | 4.839 | 6.508 | 44.041 | 10.642 | 11.26 | 107.4573632 |
| DDX6 | 8.436 | 6.7 | 94.004 | 9.567 | 8.587 | 144.6483509 |
| DDX6' | 4.032 | 4.264 | 72.482 | 7.386 | 5.188 | 139.7294297 |
| DECR2 | 4.516 | 4.226 | 70.731 | 4.591 | 3.8 | 88.5559854 |
| DHODH | 4.274 | 4.154 | 22.131 | 5.759 | 6.698 | 41.65744186 |
| DLG3 | 3.542 | 3.466 | 30.882 | 8.494 | 8.283 | 92.09288511 |
| DNM2 | 5.725 | 6.168 | 126.54 | 4.972 | 5.041 | 139.196149 |
| DOK1 | 5.02 | 4.229 | 26.589 | 5.869 | 4.663 | 39.05460251 |
| DTX2 | 4.908 | 4.947 | 89.325 | 4.133 | 3.905 | 95.83605651 |
| ECI2 | 4.797 | 4.362 | 74.831 | 5.829 | 4.892 | 112.8248373 |
| EIF4G3 | 9.371 | 8.601 | 54.294 | 5.926 | 5.289 | 44.53566096 |
| EIF4H | 7.272 | 6.819 | 73.479 | 9.385 | 9.206 | 124.024529 |
| ELAVL1 | 4.29 | 4.565 | 68.569 | 5.039 | 5.012 | 100.4525292 |
| ELAVL2 | 4.055 | 3.853 | 69.467 | 3.341 | 3.931 | 83.7703665 |
| ELAVL4 | 5.272 | 5.09 | 77.625 | 5.75 | 5.428 | 108.1893381 |
| ELN | 4.772 | 4.446 | 96.071 | 3.79 | 3.471 | 99.99294118 |
| ENAH | 5.272 | 5.027 | 71.968 | 5.9 | 5.926 | 106.4537018 |
| EVL | 6.925 | 6.72 | 107.089 | 5.671 | 6.187 | 121.0803262 |
| EWSR1 | 10.619 | 11.753 | 153.649 | 10.331 | 9.521 | 176.0506879 |
| F2 | 5.405 | 5.129 | 41.45 | 4.2 | 3.862 | 41.83593657 |
| FAAH2 | 5.773 | 5.544 | 31.196 | 3.942 | 4.071 | 29.21844161 |
| FAM103A1 | 8.503 | 8.848 | 104.286 | 9.32 | 9.586 | 146.1144024 |
| FAM120B | 5.309 | 4.062 | 26.929 | 7.035 | 7.265 | 52.27258396 |
| FAM49B | 9.628 | 9.682 | 108.243 | 7.469 | 7.798 | 111.1407019 |
| FAM81A | 7.016 | 6.923 | 77.295 | 4.501 | 4.779 | 67.89606218 |
| FKBP1A | 5.869 | 6.163 | 49.612 | 3.728 | 3.453 | 39.57772838 |
| FOXP4 | 3.37 | 3.479 | 19.155 | 4.182 | 5.087 | 33.16249564 |
| FSCB | 5.328 | 4.891 | 59.044 | 5.299 | 3.704 | 68.00670968 |
| FSIP1 | 9.152 | 9.418 | 84.043 | 9.654 | 9.068 | 109.1661246 |
| FUBP1 | 6.173 | 7.146 | 76.886 | 3.89 | 3.48 | 56.94726643 |
| GAPDH | 9.032 | 8.985 | 56.959 | 8.772 | 8.232 | 69.42916941 |
| GBGT1 | 7.123 | 7.539 | 36.676 | 5.139 | 5.031 | 33.43545692 |
| GCLM | 9.966 | 10.54 | 47.795 | 8.7 | 8.117 | 50.79070999 |
| GMPPA | 7.892 | 7.634 | 145.374 | 6.173 | 6.083 | 149.6234093 |
| GPT2 | 4.273 | 3.754 | 34.472 | 6.619 | 5.673 | 67.08999018 |
| GSTZ1 | 4.31 | 3.883 | 40.037 | 4.911 | 4.841 | 61.38251666 |
| GTF2B | 3.175 | 3.028 | 22.538 | 4.276 | 4.236 | 39.53346119 |
| HCFC2 | 3.692 | 3.661 | 55.863 | 3.761 | 3.666 | 73.58676084 |
| HGS | 10.95 | 10.749 | 88.374 | 10.713 | 10.584 | 111.7039158 |
| HNRNPA1' | 7.821 | 8.57 | 134.846 | 6.407 | 6.353 | 136.8191927 |
| HNRNPA1 | 4.71 | 4.201 | 60.366 | 3.292 | 3.281 | 59.22316058 |
| HNRNPC | 3.952 | 3.863 | 20.646 | 3.942 | 3.99 | 27.29010554 |
| HNRNPC''' | 3.592 | 3.525 | 18.887 | 3.403 | 3.181 | 22.96654265 |
| HNRNPC'' | 4.619 | 4.805 | 30.165 | 3.236 | 3.259 | 27.74961144 |
| HNRNPC' | 5.69 | 7.219 | 34.732 | 4.235 | 6.201 | 36.7046917 |
| HNRNPD | 12.074 | 12.157 | 62.635 | 8.888 | 9.068 | 60.20778995 |
| HOMER3 | 8.721 | 7.077 | 124.976 | 8.087 | 6.556 | 149.9362157 |
| HTATIP2 | 9.957 | 9.237 | 51.656 | 7.663 | 7.707 | 53.70021816 |
| IDH1 | 3.579 | 3.666 | 29.164 | 5.122 | 5.163 | 52.80901396 |
| IDH1 | 9.506 | 8.214 | 42.587 | 9.32 | 8.83 | 56.19505141 |
| IGHG1 | 4.154 | 4.543 | 40.159 | 3.372 | 3.603 | 42.57909198 |
| IGHG1' | 8.24 | 6.067 | 34.184 | 4.529 | 4.829 | 29.51288719 |
| IGHG1'' | 5.085 | 5.187 | 33.828 | 3.122 | 3.139 | 27.71364366 |
| IGHG1''' | 4.36 | 3.975 | 25.067 | 4.705 | 4.642 | 36.33176359 |
| IGHG1'''' | 3.494 | 3.322 | 16.732 | 4.008 | 3.841 | 24.91037387 |
| IRF2BP1 | 8.503 | 8.107 | 41.137 | 7.165 | 8.181 | 49.17249258 |
| Irx5 | 4.396 | 3.876 | 69.831 | 4.812 | 4.91 | 105.772335 |
| ISCU | 3.186 | 3.177 | 20.178 | 5.055 | 5.054 | 40.60954984 |
| ISG20 | 12.501 | 12.958 | 156.902 | 10.727 | 10.163 | 166.3682266 |
| ITPKB | 3.047 | 3.643 | 18.148 | 4.402 | 3.019 | 26.10904926 |
| IVD | 7.517 | 6.633 | 87.779 | 6.108 | 5.382 | 92.9782357 |
| IVD | 4.6 | 3.902 | 57.08 | 6.251 | 4.431 | 92.06329857 |
| KCNAB1 | 4.643 | 4.494 | 61.934 | 3.361 | 3.34 | 60.38418079 |
| KCNAB1 | 8.207 | 8.684 | 67.202 | 8.335 | 9.193 | 89.83158246 |
| KCNAB2 | 8.261 | 9.079 | 56.772 | 8.283 | 8.548 | 71.1330492 |
| KDM1A | 4.399 | 5.053 | 24.363 | 5.7 | 6.118 | 39.09951854 |
| KHDRBS1 | 4.142 | 3.777 | 67.257 | 3.835 | 3.366 | 80.27763397 |
| KHDRBS3 | 10.368 | 10.684 | 65.119 | 9.451 | 9.586 | 76.03433225 |
| KIF23 | 3.917 | 3.848 | 59.117 | 3.364 | 3.799 | 71.53282042 |
| KLHDC9 | 7.319 | 6.322 | 36.002 | 7.447 | 7.619 | 51.1933367 |
| LAG3 | 3.681 | 3.818 | 63.651 | 3.733 | 3.334 | 78.60575947 |
| LARS2 | 5.839 | 5.477 | 56.634 | 6.43 | 6.702 | 84.55704194 |
| LNP | 6.293 | 5.544 | 37.641 | 4.798 | 4.994 | 40.73432149 |
| LOC105372481 | 4.07 | 3.915 | 24.764 | 4.697 | 4.148 | 35.49767442 |
| LONP1 | 3.495 | 4.103 | 46.573 | 3.218 | 3.662 | 55.42101753 |
| LOR | 8.716 | 9.282 | 57.553 | 7.575 | 6.702 | 59.35269042 |
| MAGEB1 | 4.999 | 4.507 | 57.049 | 3.637 | 3.018 | 53.22332362 |
| MAPK1 | 10.555 | 11.158 | 129.995 | 8.494 | 8.888 | 134.8516402 |
| MAPK3 | 3.947 | 3.515 | 32.62 | 6.399 | 6.558 | 71.53608072 |
| MBNL3 | 4.673 | 4.692 | 44.954 | 5.433 | 5.106 | 65.29269517 |
| MBNL3' | 5.438 | 4.215 | 60.219 | 4.756 | 3.974 | 71.17219642 |
| MBP | 4.641 | 5.855 | 25.532 | 6.59 | 6.902 | 42.05396529 |
| MCM7 | 6.932 | 7.772 | 55.818 | 9.795 | 9.451 | 93.48 |
| MISP | 3.59 | 4.029 | 71.477 | 3.191 | 3.515 | 82.88130736 |
| MPST | 9.194 | 8.338 | 53.907 | 8.83 | 8.948 | 70.44844739 |
| MSI2 | 4.057 | 4.753 | 74.152 | 3.517 | 3.192 | 74.93905695 |
| MTHFD1 | 10.247 | 11.448 | 99.968 | 9.385 | 9.795 | 114.1579455 |
| NABP1 | 7.104 | 6.801 | 90.353 | 6.341 | 5.23 | 97.98689014 |
| NAT6 | 9.901 | 11.087 | 78.676 | 8.948 | 9.256 | 88.23107287 |
| NCOA3 | 4.085 | 5.008 | 43.855 | 5.086 | 4.97 | 62.67224281 |
| NECAP2 | 5.189 | 5.946 | 34.904 | 5.796 | 5.645 | 46.41667688 |
| NFYC | 9.901 | 9.901 | 92.916 | 8.658 | 8.44 | 103.8282806 |
| NG_006966.3 | 7.342 | 6.852 | 125.744 | 4.629 | 4.868 | 110.9126638 |
| NTPCR | 3.613 | 3.927 | 62.961 | 5.968 | 4.801 | 114.670268 |
| NUDT16L1 | 5.089 | 4.237 | 72.979 | 4.096 | 3.44 | 77.73866446 |
| NUDT6 | 4.273 | 3.467 | 34.586 | 3.864 | 3.532 | 43.2354038 |
| NUMBL | 4.362 | 3.815 | 76.385 | 4.635 | 4.739 | 113.0338696 |
| NUPL2 | 8.261 | 8.848 | 59.68 | 7.406 | 7.575 | 67.72020965 |
| ODAM | 4.366 | 3.809 | 20.726 | 5.019 | 5.039 | 32.78024654 |
| OLA1 | 4.315 | 3.613 | 45.319 | 4.682 | 5.144 | 72.19219902 |
| OPHN1 | 3.011 | 3.177 | 20.004 | 4.116 | 4.128 | 34.12922807 |
| OVOL2 | 13.98 | 13.98 | 141.242 | 10.163 | 10.642 | 136.0406772 |
| PABPC3 | 9.773 | 7.467 | 193.073 | 9.135 | 7.555 | 241.3185896 |
| PABPC4 | 8.107 | 8.545 | 63.92 | 6.866 | 7.204 | 70.12462665 |
| PAK4 | 3.881 | 3.472 | 42.603 | 5.467 | 4.808 | 76.00254692 |
| PAK4 | 6.147 | 5.123 | 54.653 | 7.619 | 6.335 | 86.82036287 |
| PCBP4 | 5.508 | 5.557 | 59.232 | 6.085 | 5.465 | 79.96406132 |
| PDCD6 | 10.619 | 10.882 | 97.435 | 9.795 | 9.385 | 112.2496221 |
| PFKP | 7.723 | 7.289 | 47.084 | 8.532 | 8.058 | 66.9449959 |
| PNKP | 5.687 | 5.498 | 71.551 | 6.041 | 4.881 | 90.65693381 |
| POGZ | 9.957 | 10.749 | 110.075 | 7.38 | 7.47 | 102.7974468 |
| POP7 | 7.452 | 8.164 | 70.055 | 9.068 | 8.494 | 101.2749107 |
| PPP1R13L | 5.043 | 4.78 | 85.845 | 3.841 | 3.008 | 79.67773754 |
| PRAM1 | 11.598 | 11.669 | 238.704 | 8.144 | 6.667 | 198.3131996 |
| PRR30 | 4.434 | 4.685 | 56.585 | 4.046 | 4.184 | 66.84109007 |
| PRR35 | 6.964 | 6.71 | 56.461 | 6.52 | 6.371 | 68.98117235 |
| PRRC2B | 9.421 | 9.79 | 54.396 | 10.24 | 10.318 | 74.84497982 |
| PSMB4 | 3.862 | 3.993 | 38.356 | 4.582 | 4.596 | 57.80869686 |
| PSRC1 | 8.833 | 9.293 | 87.45 | 9.01 | 8.727 | 110.3804592 |
| PUF60 | 9.613 | 8.979 | 57.702 | 7.257 | 7.269 | 58.61440678 |
| PUF60' | 5.775 | 5.176 | 35.055 | 3.854 | 3.449 | 31.09829919 |
| PUF60'' | 7.078 | 7.438 | 72.093 | 3.558 | 3.033 | 44.33000847 |
| PXK | 4.564 | 4.045 | 26.679 | 8.584 | 5.782 | 56.39552913 |
| PYCRL | 5.262 | 4.764 | 43.362 | 4.451 | 3.703 | 46.37427613 |
| QARS | 3.074 | 4.221 | 18.516 | 3.35 | 3.504 | 22.8220401 |
| QKI | 5.599 | 5.968 | 82.051 | 3.71 | 3.376 | 67.16975867 |
| RAB2B | 5.063 | 4.598 | 40.743 | 5.606 | 5.455 | 60.12502773 |
| RAB5A | 6.044 | 5.797 | 30.359 | 8.098 | 7.683 | 51.75342894 |
| RAB5C | 8.38 | 8.3 | 49.087 | 7.447 | 7.088 | 55.46271005 |
| RALY | 3.542 | 3.657 | 20.659 | 3.318 | 3.734 | 26.46468914 |
| RBM12 | 9.682 | 9.845 | 83.315 | 3.802 | 3.834 | 43.94099426 |
| RBM3 | 6.021 | 5.372 | 59.71 | 4.406 | 3.928 | 57.64191436 |
| RBM42 | 8.716 | 8.087 | 74.025 | 5.405 | 5.09 | 60.8892 |
| RBM46 | 6.269 | 6.079 | 57.217 | 5.434 | 4.527 | 60.40710412 |
| RBM46' | 5.485 | 5.719 | 51.435 | 6.677 | 5.736 | 73.47750971 |
| RBMS1 | 5.338 | 5.594 | 106.216 | 3.692 | 3.806 | 96.71325123 |
| RBMS1' | 6.246 | 6.664 | 94.774 | 4.214 | 3.994 | 79.95291829 |
| RBMS2 | 5.439 | 5.709 | 75.3 | 4.735 | 3.969 | 77.3039713 |
| RPL30 | 4.191 | 3.41 | 25.269 | 4.674 | 3.767 | 36.32880374 |
| RPLP0 | 8.071 | 5.832 | 110.638 | 5.326 | 3.747 | 95.39209432 |
| RPP25 | 3.187 | 3.578 | 53.858 | 5.17 | 4.832 | 101.3282731 |
| RXRA | 3.703 | 3.908 | 41.198 | 3.827 | 3.416 | 51.31569637 |
| SAMD4B | 4.671 | 4.146 | 53.28 | 5.945 | 5.03 | 85.16356467 |
| SAMHD1 | 10.308 | 9.609 | 53.869 | 8.715 | 9.008 | 61.9652999 |
| SATB1 | 3.539 | 3.505 | 41.934 | 4.147 | 4.166 | 63.84959658 |
| SCEL | 7.192 | 7.214 | 103.508 | 4.304 | 4.434 | 83.24359074 |
| SDS | 8.479 | 8.777 | 81.227 | 9.884 | 8.841 | 113.3662582 |
| SF3B4 | 8.503 | 9.901 | 52.491 | 8.948 | 9.586 | 68.11264122 |
| SGK494 | 7.527 | 7.479 | 52.993 | 4.491 | 6.357 | 50.20768278 |
| SHMT1 | 7.516 | 6.587 | 47.84 | 5.83 | 5.667 | 50.86772556 |
| SIRT3 | 3.793 | 3.561 | 35.52 | 3.541 | 3.262 | 43.14394904 |
| SIRT5 | 13.553 | 12.59 | 128.426 | 11.26 | 11.746 | 145.7362105 |
| SIRT5' | 10.113 | 9.332 | 126.803 | 6.665 | 6.458 | 111.8495571 |
| SKIL | 3.14 | 4.172 | 21.317 | 4.347 | 5.378 | 36.30081473 |
| SLC25A16 | 3.451 | 3.686 | 48.713 | 3.771 | 3.719 | 66.51241183 |
| SLC30A6 | 4.921 | 4.428 | 33.789 | 4.774 | 3.976 | 41.26221725 |
| SLFN5 | 3.142 | 3.794 | 22.133 | 3.861 | 4.315 | 33.67258224 |
| SMARCAL1 | 6.3 | 6.039 | 82.543 | 6.329 | 7.87 | 122.18375 |
| SMARCE1 | 9.472 | 9.371 | 61.536 | 8.44 | 8.387 | 71.07273226 |
| SMARCE1' | 8.261 | 8.032 | 51.998 | 8.494 | 8.494 | 69.84962711 |
| SMPD1 | 4.422 | 4.955 | 76.913 | 3.287 | 3.521 | 74.22409091 |
| SOHLH2 | 5.571 | 6.03 | 28.51 | 5.165 | 5.162 | 33.0734657 |
| SORBS1 | 10.619 | 11.229 | 83.694 | 8.772 | 9.32 | 89.7014313 |
| SORBS3 | 4.794 | 5.135 | 49.212 | 3.656 | 3.122 | 44.78342759 |
| SORD | 4.591 | 4.614 | 50.743 | 4.691 | 4.775 | 67.7 |
| SOX6 | 3.297 | 3.75 | 58.121 | 3.389 | 3.94 | 78.71173427 |
| SPATC1 | 6.927 | 5.504 | 41.232 | 6.451 | 4.682 | 48.04731157 |
| SPRR4 | 3.838 | 4.387 | 23.189 | 3.225 | 5.518 | 31.9723165 |
| SRA1 | 4.489 | 4.367 | 41.338 | 3.458 | 3.36 | 42.18534793 |
| SRRT | 7.179 | 6.865 | 47.824 | 5.99 | 5.367 | 50.46653878 |
| SRXN1 | 5.002 | 4.19 | 24.679 | 7.002 | 6.66 | 46.71105323 |
| SSBP1 | 9.628 | 9.271 | 63.326 | 7.89 | 7.844 | 68.35551764 |
| SSBP2 | 11.522 | 10.188 | 71.804 | 11.077 | 10.56 | 92.11357344 |
| SSBP4 | 11.753 | 11.054 | 131.736 | 10.812 | 10.478 | 158.520384 |
| STAU2 | 4.654 | 3.731 | 33.651 | 4.469 | 4.519 | 46.74568911 |
| SULT1B1 | 3.616 | 4.256 | 24.447 | 4.922 | 4.845 | 38.984474 |
| SULT1C2 | 3.952 | 5.208 | 24.715 | 4.752 | 3.362 | 28.68671636 |
| TAF6 | 7.713 | 7.569 | 50.311 | 6.671 | 5.988 | 54.23273534 |
| TAF6' | 5.842 | 5.27 | 39.608 | 8.082 | 8.548 | 75.49912019 |
| TAF9B | 3.555 | 3.043 | 66.807 | 3.708 | 3.316 | 92.54821636 |
| TBXAS1 | 10.815 | 10.071 | 55.436 | 11.354 | 11.354 | 77.42518837 |
| TCF7L1 | 3.84 | 3.562 | 54.633 | 5.471 | 4.36 | 92.91331598 |
| TIA1 | 5.524 | 4.37 | 41.828 | 8.801 | 8.828 | 94.31032855 |
| TK1 | 6.489 | 6.123 | 41.935 | 5.772 | 5.597 | 49.16013374 |
| TLE3 | 4.485 | 4.308 | 45.679 | 5.888 | 3.951 | 66.03170213 |
| TMEM116 | 5.597 | 5.479 | 28.708 | 7.308 | 6.997 | 47.49256724 |
| TRIM24 | 8.48 | 7.97 | 131.478 | 7.616 | 5.592 | 137.3897882 |
| TRMT12 | 9.321 | 10.188 | 47.579 | 9.32 | 9.385 | 58.842 |
| TRMT12' | 12.074 | 12.864 | 84.117 | 10.012 | 10.56 | 89.67654311 |
| TRMT2A | 5.389 | 5.68 | 32.421 | 6.792 | 6.509 | 50.05354331 |
| TST | 5.812 | 4.785 | 27.887 | 9.395 | 8.551 | 59.89213665 |
| TTC9 | 7.816 | 8.258 | 51.207 | 9.586 | 9.866 | 79.46002451 |
| TTC9C | 14.205 | 14.551 | 140.929 | 10.987 | 10.987 | 139.2434921 |
| TTLL1 | 4.924 | 5.345 | 95.199 | 4.915 | 4.282 | 111.3430357 |
| TUFM | 3.672 | 3.374 | 22.165 | 3.982 | 3.887 | 32.06446973 |
| UNG | 4.003 | 3.238 | 38.511 | 4.561 | 3.936 | 58.31978374 |
| VASP | 3.954 | 3.768 | 74.352 | 3.616 | 3.257 | 87.06342495 |
| VAT1 | 4.001 | 3.743 | 58.55 | 7.725 | 7.722 | 146.7302226 |
| WBP2NL | 10.247 | 9.79 | 72.892 | 8.44 | 9.256 | 83.22936641 |
| WIPF1 | 10.43 | 11.158 | 182.667 | 9.068 | 9.13 | 199.2201118 |
| WIPF1' | 9.079 | 9.472 | 118.497 | 8.494 | 8.715 | 142.0109683 |
| WWP2 | 10.619 | 10.492 | 106.58 | 8.888 | 9.256 | 118.4647001 |
| WWP2' | 7.886 | 6.964 | 79.59 | 8.033 | 7.324 | 106.1647152 |
| XAGE3 | 4.352 | 3.808 | 21.215 | 6.151 | 5.098 | 37.36843698 |
| XDH | 7.684 | 5.877 | 54.453 | 9.102 | 8.92 | 92.56220694 |
| XPNPEP3 | 3.873 | 3.616 | 34.261 | 4.278 | 4.883 | 53.92288395 |
| XRN2 | 4.138 | 3.965 | 78.397 | 4.18 | 3.77 | 100.3205217 |
| YAP1 | 5.974 | 4.977 | 40.657 | 3.48 | 3.318 | 33.77012341 |
| YEATS4 | 10.455 | 11.555 | 131.221 | 9.315 | 9.313 | 143.6292394 |
| YEATS4' | 10.43 | 10.815 | 123.6 | 9.822 | 10.019 | 148.8934712 |
| YEATS4'' | 6.391 | 5.984 | 111.03 | 4.776 | 4.102 | 104.9887804 |
| YEATS4''' | 9.472 | 9.271 | 76.91 | 8.44 | 7.937 | 86.98831598 |
| ZADH2 | 7.428 | 7.866 | 63.077 | 8.434 | 7.141 | 82.87622955 |
| ZFYVE1 | 4.131 | 4.495 | 73.061 | 6.025 | 5.73 | 127.2879842 |
| ZNF207 | 9.806 | 9.844 | 148.162 | 5.847 | 4.901 | 106.9728782 |
| ZNF207' | 7.509 | 8.107 | 80.767 | 8.033 | 7.575 | 104.2053675 |
| ZNF385A | 3.381 | 3.063 | 71.584 | 3.853 | 3.548 | 106.4069767 |
| ZNF385B | 3.394 | 3.917 | 60.53 | 4.061 | 3.7 | 83.49640082 |
