# Supplementary material for: Global Screening of LUBAC and OTULIN Interacting Proteins by Human Proteome Microarray
Source: Front Cell Dev Biol. 2021 Jun 28;9:686395. doi: 10.3389/fcell.2021.686395 (PMC8274477; doi:10.3389/fcell.2021.686395)
Supplement: Supplementary Table 2 — Detailed list of potential interacting proteins of LUBAC. [file Table_2.docx]

## Supplementary Table 2

Supplementary Table 2: The detailed list of potential interacting proteins detected by LUBAC alone

| Name | | Z-Score  (LUBAC) | | Z-Score  (LUBAC) | | IMean_Ratio  (LUBAC-BSA) | | Z-Score  (OTULIN) | | Z-Score  (OTULIN) | | IMean_Ratio  (OTULIN-BSA) | |
| --- | --- | --- | --- | --- | --- | --- | --- | --- | --- | --- | --- | --- | --- |
| SMRP1 | | 3.481 | | 3.388 | | 130.728 | | 2.91 | | 2.887 | | 52.73731 | |
| KJ902259 | | 4.162 | | 4.191 | | 157.285 | | 2.046 | | 1.684 | | 24.191 | |
| CACYBP | | 3.531 | | 3.398 | | 131.805 | | 2.838 | | 2.82 | | 50.60162 | |
| KCNRG | | 3.48 | | 3.485 | | 132.448 | | 1.998 | | 2.003 | | 47.84828 | |
| TFG | | 4.089 | | 4.002 | | 152.579 | | 1.092 | | 0.919 | | 9.908885 | |
| BDH2 | | 3.377 | | 3.606 | | 132.758 | | 1.854 | | 1.632 | | 21.28416 | |
| IGHG1 | | 3.761 | | 3.538 | | 138.408 | | 2.297 | | 2.616 | | 38.67353 | |
| BTRC | | 3.003 | | 3.11 | | 117.212 | | 2.86 | | 2.826 | | 37.61206 | |
| BCKDK | | 3.219 | | 3.49 | | 127.877 | | 2.192 | | 1.965 | | 39.54679 | |
| RBMY1A1 | | 3.646 | | 3.445 | | 134.708 | | 1.885 | | 1.898 | | 49.93695 | |
| SNX15 | | 3.953 | | 3.82 | | 146.913 | | 2.967 | | 2.968 | | 21.41105 | |
| CCDC71 | | 3.59 | | 3.32 | | 131.464 | | 2.072 | | 2.064 | | 45.63158 | |
| VGLL4 | | 3.425 | | 3.56 | | 132.818 | | 2.78 | | 2.656 | | 22.02706 | |
| MAPK1IP1L | | 5.615 | | 5.349 | | 203.994 | | 1.551 | | 1.286 | | 14.50579 | |
| EEF1G | | 4.537 | | 4.866 | | 176.058 | | 2.636 | | 2.413 | | 33.29433 | |
| BAG4 | | 3.121 | | 3.354 | | 123.681 | | 1.545 | | 1.524 | | 49.22314 | |
| ZNF3 | | 4.007 | | 3.836 | | 148.161 | | 2.811 | | 2.737 | | 51.52788 | |
| RPUSD2 | | 3.834 | | 4.206 | | 151.676 | | 1.861 | | 1.904 | | 16.98847 | |
| HNRNPAB | | 5.11 | | 4.595 | | 181.458 | | 2.051 | | 1.975 | | 16.57648 | |
| DNAJA3 | | 3.122 | | 3.277 | | 122.322 | | 2.573 | | 2.573 | | 52.07744 | |
| KRTAP20-1 | | 4.173 | | 3.967 | | 153.469 | | 2.44 | | 2.191 | | 17.54444 | |
| COL9A3 | | 4.007 | | 4.692 | | 163.463 | | 2.938 | | 2.846 | | 48.55765 | |
| ABHD16A | | 3.226 | | 3.016 | | 119.505 | | 2.766 | | 2.78 | | 19.24837 | |
| CELF6 | | 3.847 | | 3.825 | | 145.097 | | 2.828 | | 2.66 | | 74.04607 | |
| TTC17 | | 3.857 | | 4.761 | | 162.019 | | 2.918 | | 2.976 | | 47.95614 | |
| BAIAP2 | | 4.222 | | 3.863 | | 152.48 | | 0.84 | | 0.649 | | 16.74605 | |
| IL13 | | 6.419 | | 3.552 | | 186.218 | | 0.267 | | 0.301 | | 6.999161 | |
| SF1 | | 4.697 | | 6.205 | | 202.871 | | 1.107 | | 1.162 | | 37.70252 | |
| C1orf94 | | 3.112 | | 3.653 | | 128.868 | | 2.712 | | 2.869 | | 80.95413 | |
| SF1 | | 4.832 | | 4.164 | | 168.775 | | 2.48 | | 2.346 | | 55.17172 | |
| ECH1 | | 3.133 | | 3.142 | | 120.091 | | 2.802 | | 2.42 | | 24.57647 | |
| HTRA4 | | 3.987 | | 4.772 | | 164.526 | | 2.745 | | 2.602 | | 37.64257 | |
| CPSF4 | | 4.543 | | 4.106 | | 162.571 | | 2.725 | | 2.261 | | 26.86365 | |
| TMEM60 | | 3.427 | | 3.267 | | 127.589 | | 2.599 | | 2.848 | | 42.44907 | |
| NUP62CL | | 3.424 | | 3.31 | | 128.324 | | 2.746 | | 2.96 | | 19.64614 | |
| TIAL1 | | 4.669 | | 4.417 | | 170.385 | | 2.295 | | 2.219 | | 45.86948 | |
| IRX5 | | 3.514 | | 3.379 | | 131.154 | | 2.468 | | 2.888 | | 48.79581 | |
| KHSRP | | 4.954 | | 4.273 | | 172.919 | | 2.533 | | 2.368 | | 65.70309 | |
| COL10A1 | | 4.059 | | 4.842 | | 167.079 | | 1.786 | | 1.901 | | 66.14457 | |
| SRRT | | 4.714 | | 3.648 | | 157.427 | | 2.417 | | 2.042 | | 22.97529 | |
