# Supplementary material for: Global Screening of LUBAC and OTULIN Interacting Proteins by Human Proteome Microarray
Source: Front Cell Dev Biol. 2021 Jun 28;9:686395. doi: 10.3389/fcell.2021.686395 (PMC8274477; doi:10.3389/fcell.2021.686395)
Supplement: Supplementary Table 3 — Detailed list of potential interacting proteins of OTULIN. [file Table_3.docx]

## Supplementary Table 3

Supplementary Table 3: The detailed list of potential interacting proteins detected by OTULIN alone

| Name | Z-Score  (LUBAC) | Z-Score  (LUBAC) | IMean_Ratio  (LUBAC-BSA) | Z-Score  (OTULIN) | Z-Score  (OTULIN) | IMean_Ratio  (OTULIN-BSA) |
| --- | --- | --- | --- | --- | --- | --- |
| TBCE | 2.090 | 2.541 | 16.361 | 5.104 | 4.306 | 41.28983 |
| CRIP2 | 1.807 | 1.890 | 26.538 | 3.753 | 3.511 | 64.5069 |
| MRPS11 | 2.568 | 2.173 | 31.186 | 3.704 | 3.646 | 61.19845 |
| NOSIP | 1.190 | 1.355 | 23.298 | 4.297 | 3.447 | 83.31543 |
| SNX3 | 2.470 | 2.625 | 26.909 | 5.243 | 4.966 | 67.19324 |
| EFHC1 | 1.812 | 1.432 | 18.856 | 3.512 | 3.529 | 50.02285 |
| ZMAT4 | 1.494 | 1.595 | 29.990 | 3.292 | 3.258 | 77.74044 |
| SSX3 | 2.373 | 1.470 | 16.041 | 4.898 | 3.728 | 44.18579 |
| HNRNPK | 2.436 | 2.536 | 40.712 | 4.412 | 4.467 | 91.17258 |
| DPH2 | -0.081 | -0.016 | 6.061 | 4.565 | 4.831 | 226.3788 |
| LYPLA2 | 1.377 | 1.350 | 13.887 | 3.329 | 3.425 | 41.24333 |
| PDZD7 | 1.367 | 1.318 | 33.901 | 3.337 | 3.56 | 104.0454 |
| GUK1 | 1.400 | 1.428 | 12.439 | 4.086 | 3.575 | 40.22902 |
| CPTP_frag | 2.881 | 2.723 | 16.576 | 5.043 | 3.866 | 33.34478 |
| GAGE2D | 2.826 | 2.536 | 15.362 | 3.679 | 4.015 | 28.08479 |
| GMDS | 2.109 | 2.229 | 11.118 | 3.639 | 3.606 | 23.34626 |
| CBR3 | 2.163 | 2.400 | 16.531 | 4.069 | 4.018 | 36.71 |
| DCXR | 2.907 | 2.586 | 23.328 | 3.466 | 3.279 | 36.93381 |
| TRMT6 | 2.892 | 2.828 | 35.526 | 3.501 | 3.402 | 55.34563 |
| GALK1 | 1.271 | 1.255 | 12.982 | 3.056 | 3.221 | 38.51007 |
| SNX8 | 2.767 | 2.667 | 14.943 | 3.235 | 3.218 | 22.95231 |
| C11orf16 | 1.998 | 2.050 | 15.018 | 3.397 | 3.285 | 31.1619 |
| BIN3 | 1.832 | 1.562 | 10.092 | 4.153 | 4.205 | 30.15818 |
| ZFYVE19 | 1.731 | 1.793 | 31.337 | 3.181 | 3.449 | 73.09924 |
| SOCS3 | 2.280 | 2.119 | 16.755 | 3.177 | 3.354 | 31.58316 |
| DCX | 2.566 | 2.181 | 42.998 | 3.893 | 4.16 | 91.73279 |
| PKM’ | 2.932 | 2.903 | 27.324 | 4.951 | 4.065 | 53.54295 |
| AURKA | 2.697 | 2.607 | 52.084 | 3.785 | 3.47 | 91.09775 |
| F7 | 2.612 | 2.940 | 18.032 | 4.264 | 4.276 | 35.1705 |
| MARVELD2 | 1.778 | 1.850 | 16.371 | 3.307 | 3.41 | 37.67421 |
| MTMR2 | 2.320 | 2.334 | 25.349 | 3.559 | 3.133 | 46.41047 |
| NDUFAF2 | 1.981 | 1.911 | 14.412 | 3.942 | 3.989 | 36.29248 |
| GLRX5 | 2.304 | 1.766 | 11.601 | 4.97 | 4.29 | 32.43596 |
| TAF9 | 2.411 | 2.724 | 24.096 | 3.304 | 3.39 | 40.32963 |
| ADGRE1 | 2.837 | 2.478 | 33.085 | 4.089 | 3.766 | 62.16248 |
| MMP19 | 2.366 | 2.130 | 40.996 | 3.114 | 3.184 | 73.28745 |
| HSF1 | 2.793 | 2.506 | 19.403 | 3.747 | 3.879 | 35.56268 |
| ANKRD16 | 1.570 | 1.929 | 15.444 | 3.211 | 3.161 | 34.96605 |
| BOD1 | 1.273 | 1.751 | 10.148 | 3.314 | 3.336 | 27.17052 |
| TPH1 | 2.556 | 2.181 | 13.665 | 3.139 | 3.199 | 23.42523 |
| IMPDH2 | 2.423 | 2.546 | 16.566 | 3.525 | 3.018 | 27.98067 |
| GALE | 2.165 | 2.093 | 21.522 | 4.702 | 4.469 | 57.24099 |
| ANKRD10 | 2.832 | 2.315 | 42.371 | 3.813 | 3.076 | 72.63544 |
| HAO1 | 1.988 | 1.962 | 26.921 | 3.762 | 3.505 | 61.70111 |
| AKR7A3 | 2.667 | 2.635 | 45.130 | 3.191 | 3.007 | 68.34118 |
| CTF1 | 2.617 | 2.563 | 35.149 | 3.052 | 3.224 | 55.00809 |
| PSAT1 | 2.949 | 2.857 | 19.589 | 3.781 | 3.743 | 32.58813 |
| C4BPB | 2.375 | 2.111 | 31.097 | 3.383 | 3.488 | 60.32013 |
| MEF2B | 2.908 | 2.524 | 34.541 | 3.865 | 3.188 | 57.57645 |
| SNTA1 | 0.361 | 0.386 | 7.488 | 8.787 | 8.734 | 146.346 |
| ZNF830 | 2.302 | 2.062 | 29.173 | 4.48 | 3.7 | 68.17284 |
| RHPN1 | 1.744 | 1.709 | 16.543 | 7.707 | 7.937 | 88.24871 |
| CTNS | 1.629 | 1.818 | 20.424 | 3.345 | 3.365 | 49.13466 |
| RAB6B | 1.963 | 2.160 | 17.694 | 3.42 | 3.516 | 37.37495 |
| GARS | 2.201 | 2.263 | 25.590 | 3.296 | 3.063 | 46.45093 |
| TTC9B | 2.567 | 2.133 | 36.481 | 3.89 | 3.171 | 69.54189 |
| GANAB | 0.421 | 0.468 | 10.443 | 3.213 | 3.003 | 68.55277 |
| PKM | 2.695 | 2.444 | 27.324 | 4.697 | 4.079 | 58.70935 |
| PHGDH | 2.689 | 2.651 | 16.156 | 4.244 | 4.728 | 34.20765 |
| SYNJ2BP | 0.458 | 0.573 | 10.135 | 8.48 | 7.753 | 148.4121 |
| RMI2 | 1.376 | 2.035 | 15.612 | 3.346 | 3.227 | 37.20391 |
| ABHD5 | 2.331 | 1.866 | 14.537 | 4.769 | 4.336 | 38.90189 |
| COL4A3 | 2.211 | 1.671 | 30.012 | 4.013 | 3.294 | 70.21165 |
| FANK1 | 1.551 | 1.400 | 21.075 | 3.63 | 3.156 | 58.73483 |
| GAGE10 | 1.183 | 1.016 | 6.554 | 4.646 | 3.743 | 28.55486 |
| TCP11L1 | 2.195 | 2.295 | 15.690 | 3.557 | 3.78 | 32.30248 |
| ECHDC1 | 1.883 | 1.712 | 9.459 | 3.302 | 3.394 | 21.87535 |
| MARK4 | 1.850 | 1.627 | 16.012 | 4.839 | 4.212 | 50.46023 |
| PUS3 | 2.543 | 1.905 | 16.700 | 3.412 | 3.028 | 30.76268 |
| XAGE2 | 2.828 | 2.173 | 21.716 | 8.749 | 7.29 | 84.88592 |
| TGIF2LY | 1.484 | 1.376 | 16.339 | 4.123 | 3.379 | 51.31344 |
| EXOC7 | 2.932 | 2.263 | 21.175 | 4.604 | 3.596 | 42.2893 |
| SHANK2 | 2.230 | 2.008 | 33.089 | 9.586 | 8.715 | 170.8918 |
